# The Impact of Pathogens on Sepsis Prevalence and Outcome

**DOI:** 10.3390/pathogens13010089

**Published:** 2024-01-20

**Authors:** Birte Dyck, Matthias Unterberg, Michael Adamzik, Björn Koos

**Affiliations:** Klinik für Anästhesiologie, Intensivmedizin und Schmerztherapie, Universitätsklinikum Knappschaftskrankenhaus Bochum, 44801 Bochum, Germany; birte.dyck@rub.de (B.D.);

**Keywords:** sepsis, bacteria, virus, COVID-19, immunity

## Abstract

Sepsis, a severe global healthcare challenge, is characterized by significant morbidity and mortality. The 2016 redefinition by the Third International Consensus Definitions Task Force emphasizes its complexity as a “life-threatening organ dysfunction caused by a dysregulated host response to infection”. Bacterial pathogens, historically dominant, exhibit geographic variations, influencing healthcare strategies. The intricate dynamics of bacterial immunity involve recognizing pathogen-associated molecular patterns, triggering innate immune responses and inflammatory cascades. Dysregulation leads to immunothrombosis, disseminated intravascular coagulation, and mitochondrial dysfunction, contributing to the septic state. Viral sepsis, historically less prevalent, saw a paradigm shift during the COVID-19 pandemic, underscoring the need to understand the immunological response. Retinoic acid-inducible gene I-like receptors and Toll-like receptors play pivotal roles, and the cytokine storm in COVID-19 differs from bacterial sepsis. Latent viruses like human cytomegalovirus impact sepsis by reactivating during the immunosuppressive phases. Challenges in sepsis management include rapid pathogen identification, antibiotic resistance monitoring, and balancing therapy beyond antibiotics. This review highlights the evolving sepsis landscape, emphasizing the need for pathogen-specific therapeutic developments in a dynamic and heterogeneous clinical setting.

## 1. Introduction

Sepsis is a severe medical condition and a critical global healthcare issue. Worldwide, sepsis affects an estimated 48.9 million people annually and is considered to be responsible for 20% of all deaths [1]. The mortality remains high, with 20–30% of patients dying in the acute phase [1], indicating that sepsis remains one of the leading causes of death worldwide. However, even after surviving the initial phase of sepsis, patients still face an elevated risk of mortality after being discharged from hospital, with about 30% dying in the first year [2]. Treatment options are still extremely limited, with early antibiotic treatment and surgical or interventional focus control wherever possible still being the only causative therapy [3]. The main problem is the high heterogeneity of sepsis. In fact, it is now widely recognized that sepsis encompasses several phenotypes, which are rooted in the biological and clinical heterogeneity of the affected individuals [4] as well as the heterogeneity of the infected organs and the pathogens involved. Factors such as age, genetics, underlying comorbidities, concurrent injuries (including surgery), medications, and the source of infection exert significant influence on outcome [5]. Additionally, healthcare crises can also impact the mortality of sepsis [6]. In recent years, preclinical studies have increasingly emphasized the host–pathogen interactions in sepsis leading to this heterogeneity. However, the underlying pathophysiological and molecular mechanisms still remain incompletely understood.

In 2016, the Third International Consensus Definitions Task Force introduced the Sepsis-3 definition, redefining sepsis as a “life-threatening organ dysfunction caused by a dysregulated host response to infection” [7]. The new definition underscores the nonlinear pathology of sepsis and focuses on the metabolic changes the immunological syndrome elicits in the host. However, this dysregulated immune reaction must be in response to an infective agent, which distinguishes sepsis from a dysregulated immune response in reaction to trauma or other non-infectious causes (so-called sterile sepsis). Therefore, the impact the pathogen has on the phenotype of sepsis, the immune reaction, and the clinical outcome should not be underestimated. The pathogens causing sepsis can generally be divided into bacteria (Gram-negative, Gram-positive, or mixed), viruses, and fungi. Until 2020, bacterial sepsis was the absolute dominating entity, with viral sepsis being very rare [8,9]. The COVID-19 pandemic has changed this significantly, as severe cases of COVID-19 can be defined as viral sepsis [10]. While we see a significant reduction of COVID-19-induced sepsis in the ICUs in post-pandemic times, it is too early to say how this will develop in the future. Therefore, we aim to give the reader an overview of the different bacterial and viral pathogens, their prevalence, and their immunity to guide research and clinical decision-making in the future.

## 2. Bacterial Pathogens and Their Effects on Sepsis

Infectious diseases, particularly sepsis, have been recognized as a significant global health challenge, contributing substantially to mortality and hospital expenditures worldwide [11].

Initially characterized as primarily activated by Gram-negative bacteria, the understanding of sepsis has evolved over the years to encompass a broader spectrum of pathogens, including Gram-positive bacteria, fungi, and viruses [8].

**Figure 1:**
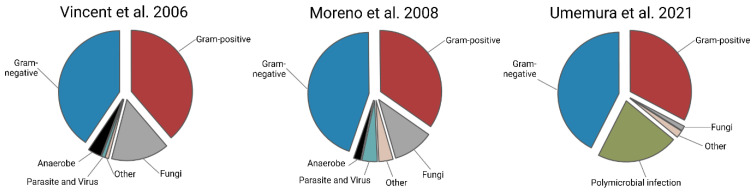
Pathogen prevalence in positive isolates in different cohorts over time, based on the publications of Vincent et al. 2006 [12], Moreno et al. 2008 [13], and Umemura et al. 2021 [8].

In their multicentric sepsis study from 2006, Vincent et al. [12] could classify about 45% of identified causal microorganisms as Gram-negative, one of the most common being *Escherichia coli* in 13% of all identified cases (Figure 1). They could also identify about 43% of their sepsis cases as caused by a Gram-positive bacterium, with *Staphylococcus* being the most common (30%). Two years later, Moreno et al. [13] showed a prevalence of about 90% of all identified infective agents in their 1099 severe sepsis and septic shock patients being bacteria. They identified about 45% as Gram-negative bacteria and about 35% as Gram-positive.

Similar levels of Gram-positive (33%) and Gram-negative (42%) sepsis were reported more recently by Umemura et al. [8] in 2021 for a Japanese cohort (Figure 1). This underscores that up to 2020, the main infective agents causing sepsis were bacteria, a fact that is mirrored by the Surviving Sepsis Campaign guidelines suggesting broad-band antibiotics in patients with sepsis or septic shock in the first hour [14]. This understanding is especially crucial as bacteremic patients have a higher mortality rate than patients with culture-negative sepsis [15].

Geographic variation in sepsis-causing pathogens is a notable aspect of infectious disease epidemiology. Sakr et al. [9] conducted a multicentric study, revealing distinct regional differences in the types of organisms responsible for sepsis. *Acinetobacter*, a Gram-negative bacterium prevalent in sewage water, exhibited significant discrepancies in infection rates across different geographical locations, with a noteworthy correlation observed between prevalence and government healthcare expenditure. This underscores the influence of regional factors on the distribution of sepsis pathogens. Moreover, Umemura et al. [8] emphasized the importance of considering geographical variations in their nationwide cohort study in Japan, noting changes in the spectrum of causative pathogens over the years. These findings highlight the need for a nuanced understanding of sepsis epidemiology, taking into account regional differences in pathogen prevalence and their potential impact on healthcare strategies and resource allocation.

Understanding how the prevalence of different bacteria evolves across regions is essential for devising targeted strategies in the face of changing epidemiological landscapes. As pathogens exhibit diverse resistance patterns and geographical disparities, the host’s immune system plays a crucial role in determining the outcomes of sepsis. Transitioning from the regional influences on pathogen prevalence, we can delve into the intricate dynamics of bacterial immunity and the host response.

All pathogens in sepsis share the commonality of entering the host and subsequently infiltrating the bloodstream. Upon entry into the human organism, pathogens are recognized through receptors. Typically, the immune system identifies pathogen-associated molecular patterns (PAMPs) of invading pathogens or damage-associated molecular patterns (DAMPs) of dying or damaged cells through pattern-recognition receptors. The family of pattern-recognition receptors (PRRs) includes, among others, the Toll-like receptor family (TLR) as well as the leucine-rich repeat (LRR)-containing protein family (NLRP). Gram-positive and Gram-negative bacteria and their specific PAMPs are recognized by different PRRs. For example, while TLR4 recognizes lipopolysaccharides (LPS) from Gram-negative bacteria, TLR2 primarily recognizes the cell wall components of Gram-positive bacteria and peptidoglycan of *S. aureus* [16].

**Figure 2:**
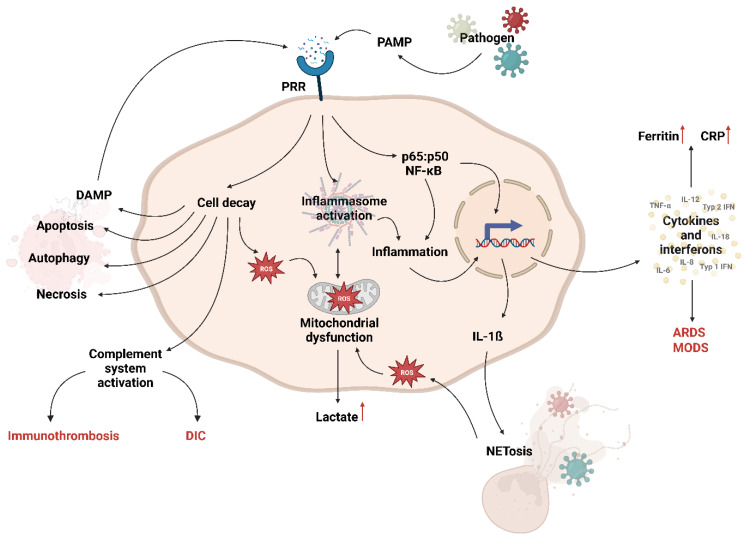
Simplified immune response during sepsis upon infection with a pathogen. Created with BioRender.com. PAMP = pathogen-associated molecular pattern; PRR = pattern-recognition receptor; DAMP = damage-associated molecular pattern; ROS = reactive oxygen species; NETosis = neutrophil extracellular trap formation; DIC = disseminated intravascular coagulation; ARDS = Acute Respiratory Distress Syndrome; MODS = Multiple Organ Dysfunction Syndrome; CRP = C-reactive protein, red upward arrow indicate elevated concentration.

The recognition of PAMPs triggers the innate immune system (Figure 2). This is the quick-reacting but unspecific arm of the immune response. PAMPs prompt monocytes and macrophages to activate the p65:p50 NF-κB translocation into the nucleus. Crucial cytokines for the pro-inflammatory response include tumor necrosis factor alpha (TNF-α), pro-interleukin (pro-IL)-1β, IL-6, IL-8, IL-12, and pro-IL-18 [17]. Furthermore, other important molecules, such as ferritin and C-reactive protein (CRP), are produced. CRP is subsequently used to opsonize extracellular pathogens, activating the complement system. In addition, the complement system is initiated by immunoglobulins in response to invading pathogens or DAMPs, leading to the release of anaphylatoxins, particularly C3a and C5a. While the activation of the complement system is crucial for protective immunity, excessive activation can result in further tissue damage and organ failure due to immunothrombosis or disseminated intravascular coagulation (DIC, Figure 2) [17]. In addition to PAMPs activating the NF-κB axis, the induction of the inflammasome is also possible. The activation of the inflammasome triggers caspase 1 to work, which results in the cleavage of pro-IL-18 and pro-IL-1β, yielding their active forms. These are secreted, and particularly IL-1β is used to recruit and activate neutrophils. These specialized cells employ a process called “NETosis” (neutrophil extracellular trap formation, Figure 2), which involves the release of extracellular structures known as neutrophil extracellular traps (NETs) [18]. These NETs can trap and immobilize bacteria, preventing their spread and providing a scaffold for antimicrobial components to exert their effects and further activate the complement system [18]. In addition, neutrophils produce vast amounts of reactive oxygen species (ROS) and nitric oxide (NO) to combat the invading pathogen [19]. In the dysregulated, septic immune response, ROS is thought to be generated in excessive amounts, leading to a range of maladaptive changes in the host. As immune cells shift towards pro-inflammation, their modes of generating energy shift as well. The cells rely mostly on glycolysis, which is increased in the pro-inflammatory phase. Along with glycolysis, the pentose phosphate pathway is increased, which triggers the production of more ROS via NOX (NADPH oxidase) and NO via NOS (nitric oxide synthase) [20]. The pyruvate generated during glycolysis is not fed into the citric cycle in the mitochondria but instead converted into lactate and released into the bloodstream. The cell does this in order to generate energy fast rather than efficiently [21]. The mitochondria are shut down, probably to protect them from excessive ROS and NO. If the host does not recover from this adaptive process, septic mitochondrial dysfunction can develop. This is one of the main differences between a regulated immune response and the septic state. The mitochondrial dysfunction is also connected to the inflammasome activation and vice versa [22,23]. Subsequently, the adaptive immune response is activated by antigen-presenting cells.

In response to the activation of pro-inflammatory networks, the anti-inflammatory response is triggered. Anti-inflammatory cytokines like IL-4, IL-10, and transforming growth factor beta (TGF-β) are, in turn, released [24]. The number of human leukocyte antigen (HLA) antigen-presenting receptors on monocytes and the cytokine response of monocytes to stimulation are decreased, and the lymphocytes are reduced by means of apoptosis [25]. As stated above, sepsis is characterized by an imbalance of the immune response, resulting in the activation of both pro- and anti-inflammatory signaling networks at the same time. Hence, the immune system is not able to restore immune homeostasis, resulting in organ dysfunctions and often in a state of immunoparalysis involving innate and adaptive immune responses [26,27,28].

## 3. Viral Pathogens and their Role in Sepsis

While the majority of causative pathogens for sepsis up to 2020 were bacteria, virus-induced sepsis has always been known. The most common virus capable of inducing sepsis in adult patients living in developed countries was the Influenza virus, with incidences ranging between 1% and almost 4% [8,9]. Especially in tropical countries, outbreaks of zoonotic viruses such as Ebola, Lassa, Marburg, Hanta, or Dengue virus can be much more prevalent [29,30]. All this, of course, was turned on its head when the COVID-19 pandemic hit in 2020. Between 2020 and 2023, the incidence of viral sepsis was more than 15% [31], peaking at 80% [32] of all sepsis cases being COVID-19-induced. 

The immunologic response to a viral infection is generally very similar to the bacterial response. For viruses, specific PRRs detecting the invading virus are known. One of the key sensors is the family of the retinoic acid-inducible gene I (RIG-I)-like receptors (RLRs) [33]. They can recognize virus-derived RNA mediating the transcriptional induction of type I interferons. As they are also triggered by host-derived RNA, the vicious cycle of reactivating the PRRs upon DAMP production is started as well [34]. The induction of type 1 interferons potentiates both the innate and adaptive immune responses to clear viral infections [35]. The pathogenic virus can also be detected by TLR3, TLR4, and TLR7, leading to the p65:p50 NF-κB translocation and the subsequent production of pro-inflammatory cytokines. In the case of COVID-19, this so-called cytokine storm was extensively discussed in the early phases of the pandemic. By now, we know that the levels of IL-6 in COVID-19 sepsis are only moderate in comparison to polymicrobial sepsis [36,37]. Furthermore, the dynamics of IL-6 seem to be fundamentally different in COVID-19 sepsis, as this cytokine increases in concentration 2–3 weeks after disease onset [36]. The same holds true for the comparison of COVID-19 with other viral sepsis forms, such as Influenza. COVID-19-induced sepsis shows a longer incubation time, delayed onset of symptoms, and a longer inflammatory phase [37]. During viral infection, the immunomodulatory effects of type 1 and type 2 interferons play a pivotal role, acting as tipping point proteins that activate subsequent immune responses. When we compare the interferon (IFN) response between COVID-19- and Influenza-induced sepsis, we find that IFN is released later during the course of severe COVID-19 (following the pro-inflammatory phase) and is less pronounced, leading to longer disease and higher severity [37].

In addition to viruses such as SARS-CoV-2 and Influenza causing sepsis, there is a significant effort to evaluate the impact virus reactivation has on survival in polymicrobial sepsis patients. The reactivation of human cytomegalovirus (HCMV) is especially widely discussed as an independent risk factor for bacterial [38] as well as for viral sepsis [39]. HCMV belongs to the family of herpes viruses and stays in the body of the host after initial infection in a latent form [40]. It can reactivate regularly during the lifetime of the host [41], which does not cause clinical symptoms and often goes undetected in immunocompetent individuals. In immunocompromised states such as sepsis, the reactivation of HCMV and other herpes viruses [42] can cause severe complications, including death. However, in recent work, we have shown that even the presence of the latent form of the virus, without reactivation, could change the immunity of patients, leaving them more vulnerable to sepsis-related death [43].

## 4. Therapeutic Approaches and Management

An effective and, above all, rapid sepsis therapy is crucial for survival [44]. For adequate antimicrobial therapy to begin, however, the causative pathogen needs to be identified rapidly. Blood cultures, therefore, must be obtained very early on and, if possible, prior to antibiotic administration. During the time till microbial identification, a “hit hard and early” administration of broad-spectrum antibiotics is used. The requirement for an exact and precise determination of pathogens is crucial, as the administration of antibiotics in the case of viral and fungal sepsis proves ineffective and may be associated with adverse effects. Conversely, for bacterial sepsis patients, every hour without appropriate antibiotic treatment correlates with an approximate 9% increase in in-hospital mortality [45]. Further, the excessive use of antibiotics is highly associated with antibiotic-resistant bacteria. Being aware of the prevalence of pathogens is crucial for monitoring the development of resistance and the spread of unknown bacteria.

If a pathogen has been identified, and the sensitivity to anti-infectives is known, the initial anti-infective therapy should be adjusted to optimize efficacy and reduce the induction of bacterial resistance. It is also suggested that in cases of clinical improvement within the first 72 h, even without pathogen identification, initial combination therapy should be de-escalated to monotherapy [46]. The reason lies in the increasing emergence of antibiotic resistance in recent times [47]. In addition, continuation of antibiotic treatment should be guided by the use of clinical biomarkers such as procalcitonin (PCT) [48].

Furthermore, the Deutsche Sepsis Gesellschaft e. V. (DSG) recommends considering additional antifungal or antiviral therapy in high-risk patients, following the focus-related guidelines [46]. With the advent of COVID-19, antiviral therapeutic approaches have moved more into the clinical focus of sepsis therapy. The aim is to inhibit viral replication directly or indirectly by interacting with key proteins of the viral replication cycle or other structures necessary for viral proliferation. The S3 guideline recommended the administration of, for example, Remdesivir in patients with COVID-19 and risk factors for a severe course [49]. Remdesivir is a direct-acting nucleotide prodrug inhibitor of the SARS-CoV-2 RNA-dependent RNA polymerase [50]. In addition, HCMV inhibitors, such as Letermovir, are routinely used in immunocompromised HCMV seropositive patients [51].

One of the greatest issues in sepsis therapy is the detection of the pathogen. In all of the studies, the percentage of unidentified pathogens remains high, ranging between 20% and 30% [8,52]. This is due to pathogens not proliferating in the blood cultures, which is considered the gold standard for diagnosing bloodstream infections. However, it is also important to acknowledge that up to 50% of blood cultures may generate false-positive outcomes due to contaminations [53]. The method is limited by their low sensitivity and long incubation time (≥72 h) and can only detect fungi or bacteria [54]. For a comprehensive viral classification, measuring nucleic acids or protein biomarkers is necessary [55]. Applications like next-generation sequencing (NGS) for nucleic acid sequences are often impractical in many hospitals due to infrastructure and cost considerations [56]. Rapid antigen tests or bedside tests are well-known for certain viruses and have become widely used, especially with the onset of the COVID-19 pandemic, but they often lack reliability to exclude infection [57].

Novel techniques have showcased remarkable improvements, particularly in the case of MALDI-TOF MS (matrix-assisted laser desorption ionization time-of-flight mass spectrometry). This technology has been observed to enhance overall survival by 4–9%, attributable to its rapid diagnostic capabilities that accurately identify bacteria and fungi [58,59]. With the combination of PCR (Polymerase Chain Reaction), a higher diagnostic yield than blood culture could be reached [60].

Currently, sepsis therapy involves a delicate balance of probabilities. The translation of current knowledge into research models is crucial for the future development of personalized therapies.

## 5. Translational Models (Animal and Cell Culture Models)

As we have stated above, sepsis is a complex, nonlinear immunological syndrome. The sepsis-3 definition has significantly altered the pathology defined as sepsis, focusing on organ dysfunction and metabolic changes [7]. Interestingly, research models have not been adapted. This means that besides a change in the definition of what sepsis is, the models to study sepsis are still largely the same, possibly hampering the translation of results to the clinic.

Animal models are often the method of choice, with mice still being the primary model organisms for sepsis. Yet, it is pertinent to note that mice exhibit well-known discrepancies in both innate and adaptive immune responses (e.g., Toll-like receptors, T cells) [58]. This may be the reason that most breakthroughs in mouse sepsis are not successfully translated to the clinic. Porcine models have a higher level of correspondence with clinical situations and human disease progression due to their closer genetic relation to humans, resulting in more easily translatable and reproducible results [59]. Nevertheless, challenges arise from the practicalities associated with the size and intelligence of pigs, impacting housing and care. Small animals, such as mice, can reproduce more quickly, which is particularly advantageous for knockout models [59]. Furthermore, the reduction, refinement, and replacement (3R) principle would argue for smaller animal models as well [60]. In mice, the gold standard for triggering sepsis is the so-called CLP model (Cecal Ligation and Puncture) [61], a highly invasive model that contrasts with the 3R principle. However, numerous injection models are known in which substances such as LPS, bacteria, or cecal slurry are administered intraperitoneally. The main focus of these models lies on the peritoneal manifestation of sepsis (Peritonitis); urosepsis and pneumonia models are known as well but not commonly used. Fecal inoculation is the method of choice for porcine sepsis models, resulting in peritoneal manifestation as well [62]. This is very interesting, as lower respiratory tract infections have a similar frequency to the peritoneal manifestation [8], but they are not as commonly translated into research models. In conclusion, currently, there is no good animal model for sepsis research.

Cell culture, of course, is the main alternative to animal models. Researchers use either immortalized cell lines or primary immune cells, such as peripheral mononuclear blood cells (PBMCs). Immortalized cell lines have the advantage of providing reproducible and consistent results, and they are cost-effective and easy to use. In contrast to primary cells, however, these cell lines usually depict a malignant state and are commonly genetically unstable. Again, this makes them less ideal for a sepsis research model. In addition, cell lines are usually of only one cell type, reducing the immense complexity of the immune system with its cell–cell interactions among multiple cell types significantly. While this makes it easier to design experiments and interpret results, it also makes these conclusions less likely to be translatable to the clinic. Primary cells hold several advantages over cell lines. They are often a population of different cell types (e.g., PBMCs consisting of monocytes, T cells, B cells, and others), are genetically stable, and are in a physiological state, not a malignant one. However, they have one serious disadvantage: they die relatively fast, and long-term experiments are often not feasible.

Currently, the sepsis-like state in cell culture experiments is often induced using LPS, activating the TLR4 signal network and resulting in an observable inflammatory reaction in the cells. This mimics the immune activation induced by Gram-negative bacteria [16]. However, what distinguishes sepsis from a mere immune reaction to Gram-negative bacteria? The main difference probably is the organ dysfunction observed in the host. As cells, be they primary cells or cell lines, cannot manifest an organ dysfunction, proper sepsis can never be observed in cell culture experiments. However, we can use surrogate markers for said organ dysfunction, such as mitochondrial dysfunction, but other research models are urgently needed to better study the dysregulated immune reaction in sepsis as well as the cause and the pathology of the organ dysfunction.

One promising approach is the use of an organoid model, although they are still extremely rare. Organoids are stem cell-derived and form a 3D culture system allowing the mimicking of organs [63]. They come with challenges like limited maturation or differing physiology [64], but they provide an opportunity to study sepsis in a less artificial way. In recent years, the so-called ‘organ on a chip’ and ‘body on a chip’ techniques have emerged, providing the possibility to grow organoids in a controlled environment. Exploring these techniques could provide a valid and more ethically acceptable alternative to traditional animal models. Nevertheless, further work is needed to establish, adapt, and optimize organoid models for sepsis research.

## 6. Conclusions

To conclude, we can summarize three main points:

1. Sepsis remains a critical global health challenge, affecting nearly 49 million people annually and contributing to 20% of all deaths. With 20–30% of patients succumbing during the acute phase, the mortality remains unacceptably high. In addition, the risk persists even post-hospitalization, emphasizing the ongoing threat sepsis poses to global health;
2. With the exception of the COVID-19 pandemic, bacterial pathogens are the most prevalent sepsis-causing agents, with viruses only playing a minor part. However, during the immunosuppressive phase of the disease, reactivation of latent viruses can increase mortality significantly;
3. There is an urgent need for innovative approaches for translational sepsis models in order to be able to accurately study the pathomechanisms of sepsis in research.

Further work is urgently needed to identify novel immunologic targets, establish more accurate research models, and translate findings more efficiently to the clinic. Sepsis is going to continue to be a crucial research topic in intensive care medicine for the foreseeable future.

## Data Availability

Not Applicable.
